# Heat Illness and Death Among Workers — United States, 2012–2013

**Published:** 2014-08-08

**Authors:** Sheila Arbury, Brenda Jacklitsch, Opeyemi Farquah, Michael Hodgson, Glenn Lamson, Heather Martin, Audrey Profitt

**Affiliations:** 1 Office of Occupational Health Nursing, Occupational Safety and Health Administration (OSHA); 2 Education and Information Division, National Institute for Occupational Safety and Health, CDC; 3 Office of Science and Technology Assessment, OSHA; 4 Office of Occupational Medicine, OSHA; 5 Salt Lake Technical Center, Directorate of Technical Support and Emergency Management, OSHA; 6 Office of Health Enforcement, OSHA

Exposure to heat and hot environments puts workers at risk for heat stress, which can result in heat illnesses and death. This report describes findings from a review of 2012–2013 Occupational Safety and Health Administration (OSHA) federal enforcement cases (i.e., inspections) resulting in citations under paragraph 5(a)(1), the “general duty clause” of the Occupational Safety and Health Act of 1970. That clause requires that each employer “furnish to each of his employees employment and a place of employment which are free from recognized hazards that are causing or are likely to cause death or serious physical harm to his employees” (1). Because OSHA has not issued a heat standard, it must use 5(a)(1) citations in cases of heat illness or death to enforce employers’ obligations to provide a safe and healthy workplace. During the 2-year period reviewed, 20 cases of heat illness or death were cited for federal enforcement under paragraph 5(a)(1) among 18 private employers and two federal agencies. In 13 cases, a worker died from heat exposure, and in seven cases, two or more employees experienced symptoms of heat illness. Most of the affected employees worked outdoors, and all performed heavy or moderate work, as defined by the American Conference of Governmental Industrial Hygienists (2). Nine of the deaths occurred in the first 3 days of working on the job, four of them occurring on the worker’s first day. Heat illness prevention programs at these workplaces were found to be incomplete or absent, and no provision was made for the acclimatization of new workers. Acclimatization is the result of beneficial physiologic adaptations (e.g., increased sweating efficiency and stabilization of circulation) that occur after gradually increased exposure to heat or a hot environment (3). Whenever a potential exists for workers to be exposed to heat or hot environments, employers should implement heat illness prevention programs (including acclimatization requirements) at their workplaces.

To understand the effectiveness of existing heat illness prevention campaigns and tools, OSHA convened the Heat Illness Workgroup* to conduct a systematic review of cases of occupational heat illness or death cited for federal enforcement under paragraph 5(a)(1) during 2012–2013. Cases were identified by OSHA’s Directorate of Enforcement Programs. For all cases reviewed, the workgroup established a list of program elements it considered important based on published literature and members’ professional experience (Table). These included information on local weather conditions, work processes and workload, employer heat illness prevention program elements, health outcomes, numbers of persons affected, and individual risk factors. When needed, OSHA Compliance Safety and Health Officers were consulted for case clarification.

During 2012–2013, a total of 20 cases were cited for federal enforcement under paragraph 5(a)(1). Thirteen cases involved a worker death attributed to heat exposure, and seven involved two or more workers with symptoms of heat illness. Thirteen worksites were outdoors. In eight cases, workers performed heavy work, and in 12 cases they performed moderate work per American Conference of Governmental Industrial Hygienists workload definitions (2). Seven cases occurred in indoor facilities with a local heat source, such as laundry equipment or combustion engines. The cases occurred in various workplaces, including two in solid waste collection, two in mail delivery, nine in outdoor worksites (e.g., ship repair, landscaping, roofing, and oil servicing), two in laundries, and five in indoor worksites with machinery or other heat sources. All heat illnesses and deaths occurred on days with a heat index in the range of 84.0°F–105.7°F (29.0°C–41.0°C), although those working in direct sunlight might have experienced a heat index that was up to 15.0°F (8.3°C) higher than reported (3).

Thirteen employers had not incorporated an approach to identifying heat illness risk (e.g., heat index), as described by the National Oceanic and Atmospheric Administration, into their heat illness prevention program (4). None of the employer heat illness prevention programs were complete. Twelve had no program at all, seven provided inadequate water management, and 13 failed to provide shaded rest areas. Only one of the employers used work-rest cycles (i.e., scheduled periods of rest between periods of work based on temperature, humidity, and the intensity of the work activity), and none had an acclimatization program (Table). Four of the 13 deaths occurred on the first day at work in a new job or after returning from time away from the job, three on the second day, and two on the third day; four deaths occurred among long-time employees. In the cases that involved heat illness but not a death, the number of days on the job did not appear to contribute to any of the heat-related incidents.

## Discussion

Heat-related deaths often occur in occupations in which workers are performing tasks in hot environments, causing them to build metabolic heat faster than their bodies can release heat and cool down. In North Carolina, during 2008–2010, work-related heat illnesses resulting in emergency department visits were more common than work-related emergency department visits with any other cause among persons aged 19–45 years (5). In Maricopa County, Arizona, during 2002–2009, outdoor work in construction and agriculture accounted for 35% of heat-related deaths in men (6). A total of 68 crop production worker deaths were reported in the United States during 1992–2006, resulting in an annual average death rate of 0.39 deaths per 100,000 crop workers (7). Particularly in agriculture, estimates of heat illness cases are likely to be undercounts because some surveys exclude workers on small farms (8).

Although OSHA’s Heat Illness Prevention Campaign’s core message “Water. Rest. Shade.” has been widely disseminated and reflects many similar public health messages (9), this review shows that some employers have not developed complete heat illness prevention programs. Strikingly, in the cases reviewed, the failure to support acclimatization appears to be the most common deficiency and the factor most clearly associated with death. Employers need to provide time to acclimatize for workers absent from the job for more than a few days, new employees, and those working outdoors during an extreme heat event or heat wave. Employers must ensure that all workers acclimatize to hot environments by gradually increasing duration of work in the hot environment. In addition, health care providers should be aware of the loss of acclimatization in their patients who have been out of work for a week or more and counsel them that they will need time to regain acclimatization once they return to their job. New workers and all workers returning from an absence of more than a week should begin with 20% of the usual duration of work in the hot environment on the first day, increasing incrementally by no more than 20% each subsequent day (3). During a rapid change leading to excessively hot weather or conditions such as a heat wave, even experienced workers should begin on the first day of work in excessive heat with 50% of the usual duration of work, 60% on the second day, 80% on the third, and 100% on the fourth day (9). Full acclimatization might take up to 14 days or longer to attain, depending on individual or environmental factors.

What is already known on this topic?

Exposure to heat and hot environments puts workers at risk for heat stress, which can result in heat illness and death. Guidance for prevention exists, but heat illness prevention programs are not formally implemented by most employers.

What is added by this report?

A review of 2012–2013 Occupational Safety and Health Administration federal enforcement cases (i.e., inspections) resulting in citations under paragraph 5(a)(1), the “general duty clause” of the Occupational Safety and Health Act of 1970, indicated a total of 20 cases involving heat illness and death among workers (13 cases of worker deaths and seven cases in which two or more employees experienced symptoms of heat illness). Most of the affected workers were outdoors and performing heavy or moderate work. In addition, most deaths occurred in the first 3 days of working, with four of them occurring on the worker’s first day. Many employers had no heat illness prevention program. Among those with such programs, many lacked basic program elements, such as water management, shaded rest areas, work-rest cycles, and acclimatization protocols. Employers’ failure to support acclimatization appears to be the most common deficiency and the factor most clearly associated with death.

What are the implications for public health practice?

Heat illness prevention recommendations include the provision of water and rest breaks in a shaded, cool area to employees. Guidance from regulatory and public/occupational health agencies should include acclimatization of workers as an essential element of employer heat illness prevention programs.

Employers should be aware of the importance of all elements, including acclimatization, in their heat illness prevention programs. They should be diligent about 1) designating a person to develop, implement, and manage the program, 2) monitoring the temperature (e.g., heat index and wet bulb globe temperature†) of their worksite, 3) providing water and rest breaks in a shaded, cool area, 4) acclimatizing workers by gradually increasing the exposure to heat or a hot environment, 5) modifying work schedules as necessary to reduce workers’ exposure to heat, 6) training workers on the signs and symptoms of heat illness, 7) monitoring workers for signs of heat stress, and 8) planning for emergencies and response. Guidance provided by CDC’s National Institute for Occupational Safety and Health includes information on acclimatization, work-rest schedules, adequate hydration, indices for monitoring environmental heat stress (including wet bulb globe temperature), and other recommendations that can be used for developing a heat illness prevention program (9,10).

The findings in this report are subject to at least three limitations. First, information collected retrospectively might fail to identify important elements such as individual prior acclimatization that might have been missed by OSHA Compliance Safety and Health Officers. Second, information from weather websites regarding past weather conditions relatively close to the worksite under consideration might not accurately represent conditions at the worksite itself (especially because at least one of the weather stations was more than 100 miles from the worksite) and thus might fail to identify the actual impact of weather. Finally, OSHA Compliance Safety and Health Officers performing workplace inspections might have missed program elements identified by the Heat Illness Workgroup because these elements were not part of routine information collection.

Additional information and resources regarding heat stress are available from CDC at http://www.cdc.gov/niosh/topics/heatstress and from OSHA at https://www.osha.gov/SLTC/heatillness/edresources.html.

## Figures and Tables

**TABLE:** Summary of heat illness and fatality cases cited by the Occupational Safety and Health Administration (OSHA)* — United States, 2012–2013

Case no.	Age (yrs)	Fatality	Type of employment	Temperature (heat index) at time of incident	Time employed	Overall employer program present	Employer provided water and supported use	Employer provided rest opportunities	Employer provided cool or shaded area	Work-rest cycle	Acclimatization program	Local uncontrolled heat source (indoor)	Clothing contribution
1	47	Yes	Waste collection	91.0°F, 32.8°C (93.8°F, 34.3°C)	1 day	No	No	Only on scheduled breaks	No	No	No	None	Wearing two flannel shirts
2	Unknown (multiple workers)	No	HVAC systems manufacturing	98.6°F, 37.0°C (105.5°F, 40.8°C)	Unknown	No	No	Limited breaks	No	No	No	Plant machinery, inoperable A/C	Unknown
3	47	Yes	Asphalt paving	97.0°F, 36.1°C (99.9°F, 37.7°C)	3 days	No	Yes	Scheduled and water breaks	No	No	No	Asphalt paver machine, hot asphalt	Unknown
4	39	Yes	Synthetic turf installation	91.9°F, 33.3°C (92.5°F, 33.6°C)	2 days	Yes	Yes	Scheduled breaks	No	No	No	Synthetic turf material	Unknown
5	Unknown	No	Commercial laundry	93.9°F, 34.4°C (102.1°F, 38.4°C)	Unknown	No	Yes	Scheduled breaks	Yes	Yes†	No	Irons, washers, dryers, no A/C or fans	Unknown
6	55	Yes	Mail delivery	102.0°F, 38.9°C (104.6°F, 40.3°C)	2 days	Yes	No	No	No§	No	No	None	Unknown
7	3 workers: 53; mid-30’s; 31	No	Oil field servicing	96.1°F, 35.6°C (102.0°F, 38.8°C)	Unknown	Yes	No	Minimal breaks	No	No	No	Rig engine and black steel pipe	Unknown
8	60	Yes	Roofing	82.9°F, 28.3°C (84.0°F, 28.9°C)	1 day	No	Yes	Scheduled breaks	Yes	No	No	Reflective roof surface	Wearing black clothing
9	Unknown (multiple workers)	No	Laundry	92°F, 33.3°C (100.0°F, 37.8°C)	Unknown	No	No	Scheduled breaks	No	No	No	Irons, washers, dryers, no A/C	Unknown
10	30	Yes	Oil and gas drilling	101.0°F, 38.3°C (101.7°F, 38.7°C)	2 days	No	Yes	Scheduled breaks	Yes	No	No	None	Unknown
11	31	Yes	Waste collection	91.0°F, 32.8°C (97.0°F, 36.1°C)	3 days	No	Yes	Minimal breaks	No	No	No	None	Unknown
12	36	Yes	Laying pipe	84.0°F, 28.9°C (88.0°F, 31.1°C)	1 day	Yes	Yes	Scheduled breaks	Yes	No	No	None	Unknown
13	Unknown (multiple workers)	No	Printing services	93.9°F, 34.4°C (98.6°F, 37.0°C)	Unknown	No	No	Limited breaks	No	No	No	Machinery	Unknown
14	59	Yes	Ship repair	87.1°F, 30.6°C (94.5°F, 34.7°C)	1 day	No	No	Breaks as needed	No	No	No	None	Unknown
15	45	Yes	Mail delivery	93.9°F, 34.4°C (98.6°F, 37.0°C)	>1 year	Yes	Yes	No	No	No	No	None	Unknown
16	20’s (2 workers); 35 (1 worker)	No	Roofing	97.0°F, 36.1°C (105.5°F, 40.8°C)	2 weeks (1 worker); 2–3 days (2 workers)	No	Yes	Scheduled breaks	Yes	No	No	Hot tar pots	Unknown
17	Unknown (2 workers)	No	Military post exchange	90.0°F, 32.2°C (97.9°F, 36.6°C)	>1 year	Yes	Yes	No	No	No	No	Not functional A/C, metal trailer, asphalt parking lot	Unknown
18	64	Yes	Waste handling and recycling	93.9°F, 34.4°C (100.8°F, 38.2°C)	1 year	Yes	Yes	One 45-minute break in 12-hour shift	No	No	No	Radiant heat from motors, aluminum walls	Unknown
19	68	Yes	Sauna	82.4°F, 28.0°C (82.9°F, 28.3°C)	Unknown	No	Yes	Scheduled breaks	Yes	No	No	Sauna temperature 200.0–250.0°F; (93.3–121.1°C) radiant heat from stone walls	Shirt, sweatshirt and sweat pants
20	64	Yes	Park	113.0°F, 45.0°C (105.7°F, 40.9°C)¶	>1 year	Yes	Yes	Breaks as needed	Yes	No	No	None	Unknown

**Sources:** OSHA’s Directorate of Enforcement Programs database for heat case inspections. OSHA Compliance Safety and Health Officers’ inspection records. Investigators’ interviews with Compliance Safety and Health Officers about the inspections.

**Abbreviations:** HVAC = heating, ventilation, and air conditioning; A/C = air conditioning.

* OSHA convened the Heat Illness Workgroup to conduct a systematic review of cases of occupational heat illness or death cited for federal enforcement (i.e., inspections) under paragraph 5(a)(1), the “general duty clause” of the Occupational Safety and Health Act of 1970, for the period 2012–2013. Cases were identified by OSHA’s Directorate of Enforcement Programs. For all cases reviewed, the workgroup established a list of program elements it considered important based on published literature and members’ professional experience.

† 75% laundry sorting and 25% rest.

§ A/C unavailable in mail delivery vehicles.

¶ Humidity was very low (7%), making the heat index lower than the temperature.

## Footnotes

* The Heat Illness Workgroup was created in 2014 after an informal internal review of OSHA’s Heat Illness Campaign and consisted of representatives from the various offices (all listed as coauthors in this report) involved in campaign materials development. The group invited a representative from CDC’s National Institute for Occupational Safety and Health when they became aware of the CDC/NIOSH document revision, *Criteria for a Recommended Standard: Occupational Exposure to Heat and Hot Environments* that was underway.

† Wet bulb globe temperature is the measure of heat stress in direct sunlight that takes into account temperature, humidity, wind speed, sun angle, and cloud cover. This differs from the heat index, which takes into account temperature and humidity and is calculated for shady areas. Additional information available at http://www.srh.noaa.gov/tsa/?n=wbgt
